# Assessing Key Factors Influencing Successful Resuscitation Outcomes in Out-of-Hospital Cardiac Arrest (OHCA)

**DOI:** 10.3390/jcm13237399

**Published:** 2024-12-04

**Authors:** Cristian Ichim, Vlad Pavel, Patricia Mester, Stephan Schmid, Samuel Bogdan Todor, Oana Stoia, Paula Anderco, Arne Kandulski, Martina Müller, Philipp Heumann, Adrian Boicean

**Affiliations:** 1 Faculty of Medicine, Lucian Blaga University of Sibiu, 550169 Sibiu, Romania; cristian.ichim@ulbsibiu.ro (C.I.); oana.stoia@ulbsibiu.ro (O.S.); adrian.boicean@ulbsibiu.ro (A.B.); 2 Department of Internal Medicine I, Gastroenterology, Hepatology, Endocrinology, Rheumatology and Infectious Diseases, University Hospital Regensburg, 93053 Regensburg, Germany; vlad.pavel@klinik.uni-regensburg.de (V.P.); patricia.mester@klinik.uni-regensburg.de (P.M.); stephan.schmid@klinik.uni-regensburg.de (S.S.); arne.kandulski@klinik.uni-regensburg.de (A.K.); martina.mueller-schilling@klinik.uni-regensburg.de (M.M.); phillip.heumann@ukr.de (P.H.)

**Keywords:** resuscitation, return of spontaneous circulation, out-of-hospital cardiac arrest, adrenaline

## Abstract

**Background:** Out-of-hospital cardiac arrest (OHCA) is a critical health issue with survival influenced by multiple factors. This study analyzed resuscitation outcomes at the County Clinical Emergency Hospital of Sibiu, Romania, during pre-COVID-19 and pandemic periods. **Methods:** A retrospective analysis of 508 OHCA patients (2017–2020) assessed the return of spontaneous circulation (ROSC) as the primary endpoint. Statistical methods included decision tree analysis, logistic regression and ROC curve analysis to evaluate the predictive value of adrenaline dose and patient factors. **Results:** The mortality rate was 68.7%, with non-shockable rhythms predominant among fatalities. Rural patients, though younger, had lower ROSC rates than urban counterparts. Logistic regression showed that lower adrenaline doses (≤4 mg, OR 11.835 [95% CI: 6.726–20.27]; 4–6 mg, OR 2.990 [95% CI: 1.773–5.042]) were associated with better ROSC outcomes. **Conclusions**: A multivariable model (AUC = 0.773) incorporating demographics and pandemic status outperformed adrenaline dose alone (AUC = 0.711).

## 1. Introduction

Sudden cardiac death (SCD) occurs when sudden cardiac arrest (SCA) leads to an unexpected and abrupt loss of heart function, resulting in a collapse of circulation and subsequent death [1]. The annual incidence of SCD in the US is currently estimated to range from 180,000 to 250,000 cases. Evidence suggests that, alongside the reduced mortality from coronary artery disease, there has been a marked decrease in SCD rates in the US during the latter half of the twentieth century [2]. Globally, out-of-hospital cardiac arrest (OHCA) incidence was higher in adults than in children, with a higher treatment response in North America, Australia and Europe; meanwhile, the lowest ventricular fibrillation (VF) survival rates were in Asia [3]. Over 60% of OHCAs occur either in patients for whom cardiac arrest is the first sign of an underlying condition or in patients who were previously identified as having the disease but were considered at a lower risk [4]. Research indicates that in genetically predisposed individuals, cardiac arrest and SCD can be the initial signs of coronary artery disease (CAD). In these cases, myocardial infarction or ischemia is often diagnosed [5]. The risk of SCD increases significantly with age and with men being more susceptible than women of the same age. While SCD is rare in infancy, its incidence rises dramatically with age, reaching up to 200 cases per 100,000 person–years after 70 years [6]. Population-based studies have identified several common risk factors for SCA and SCD, including those associated with atherosclerotic cardiovascular disease, left ventricular hypertrophy, and cardiac conduction abnormalities. Smoking is a significant predictor of SCD risk with a 2.5-times-higher annual incidence of SCD compared with non-smokers. Additionally, these studies indicate that individuals with structural heart diseases and atherosclerotic cardiovascular disease are at a higher risk for SCA and SCD [7,8]. Although the overall cause of SCD is primarily CAD, in younger individuals, common causes include inherited cardiac arrhythmias, inherited cardiomyopathies, myocarditis and coronary artery anomalies [9]. Less common causes of SCD include drug toxicity, coronary artery spasm and cardiac trauma [10]. VF and ventricular tachycardia (VT) were once the most common causes of OHCA. Recent studies have shown that asystole and pulseless electrical activity (PEA) are now the most frequent initial rhythms in more than half of these cases [11].

To improve survival rates, effective strategies include starting CPR (cardio-pulmonary resuscitation) promptly for witnessed cardiac arrests, encouraging bystander CPR, and ensuring rapid defibrillation [12]. Survival rates after an OHCA drop sharply within the first few minutes. Fewer than a quarter survive beyond five minutes and nearly none survive past ten minutes [13]. An important component of advanced cardiovascular life support (ACLS) is administering intravenous or intraosseous adrenaline 1 mg every 3 to 5 min, which improves the odds of successful resuscitation by enhancing coronary and cerebral perfusion pressure through α-adrenergic-mediated vasoconstriction, leading to its beneficial effects [14]. In cases of refractory ventricular fibrillation, administering amiodarone after at least three defibrillation attempts significantly increases the success rate of resuscitation compared with lidocaine or placebo [15].

We conducted a detailed analysis to identify the main factors predicting survival after resuscitation from out-of-hospital cardiac arrest (OHCA). Specifically, we examined variables such as sex, age, rural or urban location, the experience of the doctor conducting the intervention, the type of rhythm present at the time of the event and the drugs administered during resuscitation. By evaluating these factors, we aimed to pinpoint which ones most significantly impact survival rates, thereby providing insights that could inform and improve resuscitation practices and outcomes in OHCA cases.

## 2. Materials and Method

Our study was a retrospective, single-center observational analysis conducted at the County Clinical Emergency Hospital of Sibiu, Romania aimed at evaluating outcomes in patients who underwent resuscitation for out-of-hospital cardiac arrest (OHCA) from January 2017 to December 2020. This timeframe includes both the pre-COVID-19 pandemic period and the pandemic phase, facilitating a comparative analysis of resuscitation outcomes across these distinct periods. A total of 508 patients met the inclusion criteria and were included in the final analysis. Inclusion criteria required patients to be over 18 years of age, have complete identification data and provide information on the location where the OHCA occurred (urban or rural), as well as the type of initial cardiac rhythm at the onset of the arrest (shockable or non-shockable). The primary outcome of interest was the return of spontaneous circulation (ROSC), which was assessed both at the scene (pre-hospital) and upon hospitalization. ROSC was defined as the resumption of circulation, characterized by a sustained pulse, effective breathing, improved blood pressure and other signs of circulation, sustained for at least five minutes before the patient was transferred to the intensive care unit (ICU) [16]. Out of the 508 patients included in the study, 349 died during the study period, resulting in a mortality rate of 68.7%. Due to the retrospective nature of the study, informed consent was waived as it involved the analysis of pre-existing data. The data were carefully extracted from records maintained by the Institute for Emergency Situations in Sibiu County, ensuring a comprehensive and accurate dataset for our analysis.

Continuous data were summarized as median values with interquartile range (IQR) and were compared using the Mann–Whitney U test, appropriate for non-normally distributed data. Categorical data were presented as counts with percentages and analyzed using the Chi-square test to explore associations between different categories.

To further analyze the data, decision tree analysis was employed to identify key decision nodes and pathways influencing patient outcomes. This method helped in visualizing the impact of various factors on resuscitation success and mortality. Additionally, univariable and multivariable logistic regression analysis was conducted to determine the factors predicting mortality in out-of-hospital cardiac arrest (OHCA) patients. Model performance was assessed using Receiver Operating Characteristic (ROC) curve analysis. Predicted probabilities from the logistic regression models were plotted and the area under the curve (AUC) was calculated for both the adrenaline dose alone and the multivariable model. Optimal cut-off points for continuous variables like adrenaline dose were selected based on the decision tree analysis.

Odds ratios (OR) were calculated for the logistic regression analysis, with 95% confidence intervals (CI) presented to provide an estimate of the precision and reliability of the odds ratios. A significance level (alpha) of 0.05 was used for all statistical tests to determine statistical significance. Statistical analyses were performed using IBM SPSS Statistics version 26.0, ensuring a comprehensive and reliable evaluation of the data.

## 3. Results

For our analysis, we included 508 patients assessed in pre-hospital care or admitted to the ED (emergency department) for OHCA. A number of 349 patients (68.7%) succumbed, mostly presenting with non-shockable rhythm (88.4%). There were no sex differences among patients whose resuscitation was not successful (although a slight trend in male sex was seen), also there were no age differences between subjects (Table 1), but the population from rural areas was significantly younger than from urban areas [median age 65 (55–76) vs. 69 (60–80), *p* = 0.036]. Our analysis revealed that patients in rural residency had lower chances of successful resuscitation (23.5% vs. 34.1%). As expected, patients with shockable cardiac rhythm had a higher chance of successful resuscitation (Table 1). The incidence of shockable rhythms before and during the pandemic did not differ (χ^2^ = 0.617, *p* value—0.432).

We conducted a decision tree analysis to determine the key factors influencing successful resuscitation outcomes in OHCA individuals. Key variables included patient age, sex, initial rhythm, adrenaline dose, residency location and other factors like doctor’s experience and the COVID-19 pandemic.

**Figure 1:**
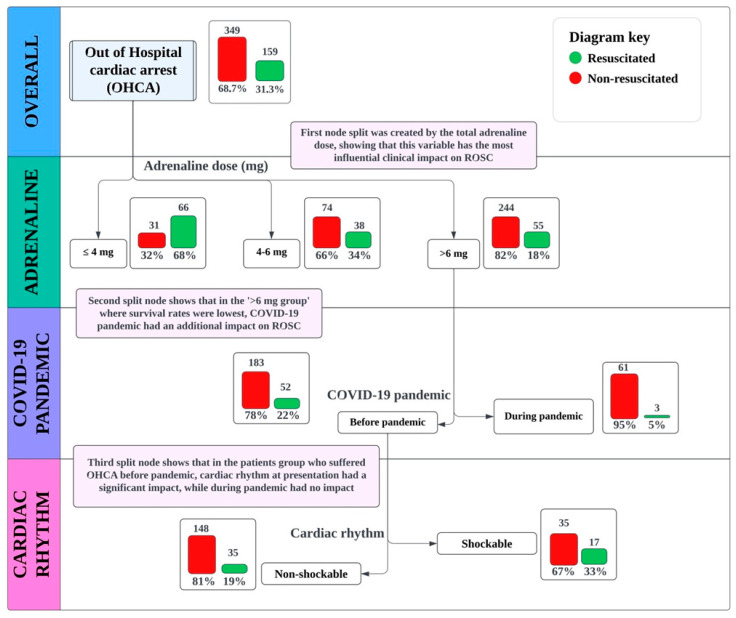
Decision tree analysis (CHAID method): key features influencing successful resuscitation are adrenaline dose, COVID-19 pandemic and the type of rhythm.

A decision tree model was developed using the CHAID (Chi-squared Automatic Interaction Detector) algorithm, which leverages the Chi-squared test to determine the optimal splits in the dataset (Figure 1). This technique focuses on enhancing the homogeneity of outcomes within each node, ensuring that the resulting subgroups are as distinct as possible in terms of the target variable. The initial split in the decision tree was determined by the dose of adrenaline administered to patients. Subsequent splits were based on whether resuscitation occurred during the COVID-19 pandemic and the type of initial heart rhythm.

Patients were then divided into three groups according to the amount of adrenaline (EPI) they received: group 1 included those who received 4 mg or less, group 2 consisted of those who received between 4 and 6 mg and group 3 comprised those who received more than 6 mg.

**Figure 2:**
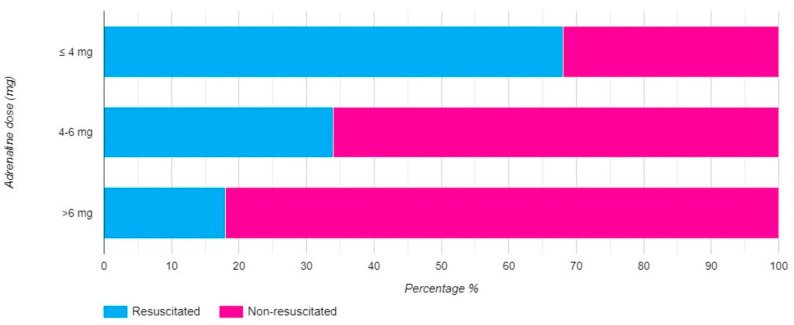
Out-of-hospital cardiac arrest success rate according to adrenaline dose (mg) given.

Further analysis involved a multivariable regression model to assess the impact of adrenaline dose on the success of resuscitation. This model showed that the dose of adrenaline, categorized into these three groups, was a significant predictor of resuscitation success. When group 3 (more than 6 mg of EPI) was used as the reference category, group 1 (4 mg or less) had an odds ratio (OR) of 11.835 (95% CI: 6.726–20.827) for successful resuscitation. Group 2 (4–6 mg) had an OR of 2.990 (95% CI: 1.773–5.042) (Figure 2). These results were adjusted for several confounding factors, including age, sex, urban residency, type o2sazaqf initial heart rhythm and whether resuscitation occurred during the COVID-19 pandemic (Table 2).

The predictive capacity of the multivariable model was evaluated by comparing the predicted probabilities for each individual, as provided by the model, to the actual outcomes. This evaluation was conducted using the Receiver Operating Characteristic (ROC) curve and the Area Under the Curve (AUC), which was calculated to quantify the model’s performance.

Additionally, the AUC of the multivariable model’s predicted probabilities was compared with the AUC derived from using the adrenaline dose (measured in mg) as the sole predictor. This comparison aimed to determine whether the multivariable model, which includes adjustments for various confounding factors (such as age, sex, urban residency, type of initial heart rhythm and resuscitation during the COVID-19 pandemic), provided a significant improvement in predictive accuracy over the use of adrenaline dose alone.

**Figure 3:**
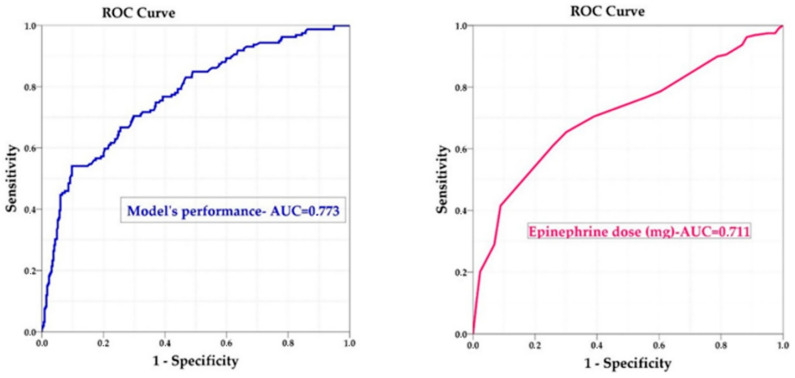
Receiver’s operating curve showing the accuracy of the multivariable model predictive probability and epinephrine does (mg) in determining the chance of a successful resuscitation.

The results demonstrated that both the multivariable model’s predicted probabilities and the adrenaline dose alone performed relatively well in predicting successful resuscitation outcomes. However, the predictive power of the adrenaline dose alone was found to be weaker compared with the multivariable model, as indicated by the AUC values. Specifically, the AUC for the adrenaline dose alone was 0.711, while the AUC for the multivariable model was higher at 0.773 (Table 3 and Figure 3).

A post hoc power analysis was conducted to evaluate whether the sample size was sufficient to detect the observed effects. With a total sample size of *N* = 508, an alpha level of *α* = 0.05 and the observed effect size (*f*2 = 0.02), the analysis yielded a power (1 − β) of 0.82. This result indicates that the study is adequately powered to detect small to medium effect sizes, meeting the conventional threshold for sufficient power (1 − β ≥ 0.80).

## 4. Discussion

This study consisted of 508 patients who suffered OHCA and were resuscitated prior to and during the first part of the COVID-19 pandemic (in 2020). A number of 349 (69.7%) patients died while the rest had a successful resuscitation. The main aim of this study was to find variables that can influence the chance of ROSC and the reliability of these independent markers in explaining the resuscitation rate.

OHCA resuscitation rates nowadays are influenced by various factors. In a study by Wang et al., age, early defibrillation, adrenaline doses, intubation and the duration of resuscitation were identified as independent predictors of return of spontaneous circulation (ROSC) [17]. Our study showed a 30% successful OHCA resuscitation rate. However, our study did not assess the survival rate at discharge. In contrast, a report by Saghafinia et al. showed that even though the success rate of CPR of in-hospital cardiac arrest (IHCA) was 30%, the survival rate at discharge was only 12% [18].

Even though previous studies showed age to be an independent variable in measuring successful resuscitation and outcome [17], our study did not show any significant association (Table 1). Recent evidence shows that age influences CPR outcome especially on vital aspects [19]. However, this should not be used as a criterion for initiating CPR, as other factors, such as frailty and resilience, may be more reliable independent variables [20]. In our research, one explanation for the lack of age difference between resuscitated and non-resuscitated cases might be the fact that patients in rural areas were significantly younger than those in urban ones (*p* = 0.036). Studies on differences between rural and urban resuscitation rates show that rurality predicts a worse outcome with lower survival rates at discharge and prolonged time-to-defibrillation [21]. In a Korean study, improvements in survival rates of OHCA during a 4-year period were seen only in urban areas with no improvements in rural ones [22]. In our study, rural areas usually extend from 5 to 10 km (the proximal ones), 10 to 30 km (the median ones) and 30 to 50 km (the distant ones). Many of the rural areas have other proximal smaller emergency centers that provide crews for CPR; therefore, the distance from the main center to the place where the OHCA occurred could not be a valid variable. Also, information regarding time to resuscitation is not available for most of the patients so they cannot be included in the analysis to check for their determinant role.

The impact of adrenaline dose on ROSC is highly controversial, making it difficult to attribute its influence directly due to the risk of resuscitation time-bias [23]. It is well established that prolonged resuscitation time negatively impacts the likelihood of ROSC [23,24]. Thus, distinguishing between the effects of adrenaline dosage and the influence of prolonged resuscitation time is challenging. Typically, 4 mg of adrenaline is administered over approximately 10–15 min within the non-shockable rhythm protocol and about 20 min within the shockable rhythm protocol [25]. Patients receiving adrenaline doses equivalent to this timeframe generally have a higher chance of resuscitation and ROSC (Figure 2). Studies on optimal resuscitation time regarding the initial rhythm suggest that 12 min is the optimal cut-off for successful resuscitation, which aligns with the interval in which 4 mg could be administered [26]. Another study indicated that administering more than 5 mg of adrenaline predicted unsuccessful prehospital resuscitation [27]. Our comparative results showed that patients receiving 4–6 mg and over 6 mg of adrenaline had lower success rates (Figure 1 and Figure 2).

The influence of adrenaline dose is difficult to separate from the influence of resuscitation time. Research indicates that the resuscitation period during which a 4 mg dose of adrenaline could be administered coincides with the period associated with positive outcomes, making it challenging to draw definitive conclusions. A study by Sigal et al. found that the early administration of adrenaline and lower cumulative doses until ROSC predict favorable outcomes at discharge. In contrast, higher doses are associated with lower survival rates and poorer neurological outcomes in survivors [28]. In our study, we managed to segregate patients into group risk according to the amount of adrenaline they received (Figure 2). When comparing patients that received more than 6 mg of adrenaline with patients receiving 4–6 mg and 4 or less, the chance of ROSC increases exponentially from 3-fold to nearly 12-fold higher (Table 2). We could state that adrenaline dose is more a time surrogate variable rather than an independent variable since it is difficult to quantify the immediate effects of adrenaline on resuscitation success and also research shows that the importance of adrenaline dose is more seen in survival than discharge rates.

The COVID-19 pandemic has significantly impacted the incidence, presentation, management, and outcomes of time-sensitive medical conditions, including cardiac arrest. During the pandemic, changes in healthcare delivery, patient behavior and system capacity have influenced how cardiac arrest cases were treated [29]. Survival and neurologic outcomes after both out-of-hospital and in-hospital cardiac arrests worsened. Contributing factors include decreased bystander CPR rates, delayed emergency response and transport times, higher incidence of non-shockable rhythms and reduced access to emergency and hospital care due to COVID-19-related hospitalizations [30]. Our study highlighted the impact of the COVID-19 pandemic on OHCA successful resuscitation rates and the results showed that, overall, the chance of ROSC in individuals resuscitated during the pandemic was halved (OR 0.494) (Table 1). Moreover, the decision tree analysis highlighted a very important distinction, meaning that the most significant impact that occurred was on patients receiving more than 6 mg of adrenaline (meaning prolonged resuscitation) (Figure 1). This result shows that, in our case, the COVID-19 pandemic had low influence on cases receiving lower doses of adrenaline (≤4 and 4–6 mg), meaning lower resuscitation times and higher chances for ROSC, but this influenced mortality rates in patients with lower chances who received over 6 mg of adrenaline, therefore amplifying the mortality of this group. Similar results were seen in a study by Ball et al. where the incidence of cardiac arrest showed no distinction regarding incidence during the COVID-19 pandemic, but due to the prolonged time until the first defibrillation or adrenaline infusion, survival rates at discharge decreased to nearly 50% (11.7% vs. 6.1%; *p* = 0.002) [31].

Regarding the initial recorded rhythm in patients with OHCA, the resuscitation rate among shockable rhythms was 74.6% (63.4–83.4%) and this variable showed a high predictive value (*p* < 0.001) (Table 2). A study in Denmark showed that initial shockable rhythm is usually associated with cardiac conditions and is associated with a higher chance of ROSC and 30 days and 1-year survival [32]. However, a decision tree analysis revealed a key finding: before the pandemic, ROSC was more likely in cases with a shockable rhythm, whereas during the pandemic, there was no significant difference in outcomes for individuals who received more than 6 mg of adrenaline (Figure 1). This could also be due to the contribution of lower promptitude of pre-hospital services. A study showed that during the pandemic, ROSC rates were lower in SARS-CoV-2 positive patients [33]. Another study showed that during COVID-19 pandemic, shockable rhythms were less common [34].

A study conducted in Turkey revealed that adrenaline use was an independent variable that predicted a lower incidence of ROSC [35]. Our research purpose was to test if adrenaline dose alone is a good predictor of ROSC in OHCA. We found that the AUC of the adrenaline dose is relatively acceptable in predicting death and the prediction becomes even more accurate when we take into account sex, age, urban residency and shockable rhythms but is still somewhat modest (Table 3 and Figure 3). Interestingly, a study in China showed that age, sex, time of CPR and adrenaline dose offer a very strong prediction model for ROSC [36]. Even though we think that factors like rurality and the COVID-19 pandemic influenced the chance for ROSC in OHCA, they offer a rather modest predictive power on the probability of ROSC and variables like time-to-defibrillation, bystander CPR, or time-to-first adrenaline dose would have been variables that could enhance the multivariable model prediction ability. Initially, we speculated that the doctor’s experience would have an impact on ROSC (Table 1); however, this correlation was not statistically significant. Interestingly, a study from Japan showed that the presence of a physician in OHCA resuscitation did not improve the ROSC rate but it was rather the contribution of factors like bystander CPR or shockable/non-shockable rhythm [37]. This proves that ROSC success is rather determined by time-dependent factors like early defibrillation or bystander CPR, proximal access to medical facilities like in urban regions, and the absence of any interfering factor that delays the time-to-CPR (like COVID-19 pandemic) and experience-related factors have little contribution.

Some limitations could be not assessing therapies with potential for alleviating arrhythmogenic burden. These therapies may influence outcomes in patients with OHCA, and their impact represents an important area for future research [38]. Another one could be a lack of information on time until the first adrenaline dose is administered or until the first shock is delivered; these could be important elements in the statistic models.

## 5. Conclusions

In conclusion, our findings indicate that the dose of adrenaline is a significant independent predictor of the likelihood of ROSC. However, it is essential to recognize that the duration of resuscitation may act as a confounding variable in this relationship. While the dose of adrenaline appears to correlate with worse outcomes, there is limited research to support that an immediate, direct impact on resuscitation success is solely attributable to the administered dose. Instead, it is likely that the cumulative dose of adrenaline reflects the prolonged duration of CPR efforts, making it a time-dependent variable rather than a straightforward predictor. This suggests that prolonged resuscitation leads to a higher cumulative dose of adrenaline, making it more difficult to interpret its direct efficacy.

Furthermore, by integrating additional factors such as rurality, the impact of the COVID-19 pandemic and the type of initial rhythm into our analysis, we were able to construct a more accurate and robust multivariable model. The COVID-19 pandemic, in particular, had a pronounced negative effect on patient outcomes, especially for those who required higher doses of adrenaline, likely due to resource strain and delays which contributed to prolonged resuscitation efforts. These findings underscore the critical need for enhanced access to timely medical care and resources, particularly in underserved rural areas of Romania, where disparities in emergency response may further compound the challenges in achieving successful resuscitation. Improving resource availability and response times in these regions could potentially reduce the need for extended CPR and high cumulative doses of adrenaline, ultimately improving patient outcomes.

## Figures and Tables

**Table 1:** General characteristics of the OHCA patients.

Variable			Out-of-Hospital Cardiac Arrest Resuscitation (OHCA)		*p*-Value
		Count *N* (%)	Non-Successful (*N* = 349; 68.7%)	Successful (*N* = 159; 31.3%)	
Demographic features					
Sex	Masculine	301 (59.3%)	238 (71.3%)	63 (36.2%)	0.085
	Feminine	207 (40.7%)	111 (53.6%)	96 (46.4%)	
Age, years			68 (59–78)	68 (55–79)	0.463
Residency	Urban	372 (73.2%)	245 (65.9%)	127 (34.1%)	0.022
	Rural	136 (26.8%)	104 (76.5%)	32 (23.5%)	
Clinical features					
Cardiac rhythm	Shockable		53 (15.2%)	43 (27.0%)	0.002
	Non-shockable		296 (88.4%)	116 (73.0%)	
NaHCO_3_-use			120 (34.4%)	63 (39.6%)	0.254
Atropine dose, mg ^‖^			0 (0–1)	0 (0–3)	0.016
Lidocaine use			9 (2.6%)	5 (3.1%)	0.718
Adrenalin dose, mg			8 (5–10)	5 (3–8)	<0.001
Amiodarone dose, mg ^†^			300 (300–450)	300 (300–450)	0.404
Miscellaneous features					
Doctor’s experience (over 15 years in ED)			262 (75.1%)	123 (77.4%)	0.577
COVID 19-Pandemic	Before		255 (73.1%)	126 (79.2%)	0.136
	During		94 (26.9%)	33 (20.8%)	

^‖^—Patients with severe bradycardia prior to cardiac arrest; ^†^—Patients with shockable rhythm; significant values are flagged with pink.

**Table 2:** Multivariable logistic regression model of variables influencing the success of OHCA resuscitation.

				Confidence Interval of 95% for OR	
Variable	B-Coef.	*p*-Value	Odds Ratio	Lower	Upper
Age	−0.015	0.031	0.985	0.971	0.999
Male Sex	−0.339	0.148	0.712	0.450	1.128
Group 1 *	2.471	<0.001	11.835	6.726	20.827
Group 2 **	1.095	<0.001	2.990	1.773	5.042
Group 3 ***	-	<0.001	-	-	-
Urban residency	0.572	0.027	1.773	1.068	2.941
Shockable rhythm	1.082	<0.001	2.949	1.729	5.030
COVID-19 pandemic	−0.706	0.008	0.494	0.293	0.833

* received 4 mg or less of adrenaline; ** received 4–6 mg of adrenaline; *** received more than 6 mg of adrenaline, significant values are flagged with pink.

**Table 3:** Area under the curve (AUC) of the multivariable model-given probability and adrenaline dose alone.

			Confidence Interval 95%	
Variables	AUC	*p*-Value	Lower	Upper
Multivariable model predictive probability	0.773	<0.001	0.728	0.817
Adrenaline dose (mg)	0.711	<0.001	0.659	0.763

## Data Availability

The datasets generated and analyzed during the current study are not publicly available due to institutional restrictions but are available from the corresponding author upon reasonable request.

## References

[1] Narayan S.M., Wang P.J., Daubert J.P. (2019). New Concepts in Sudden Cardiac Arrest to Address an Intractable Epidemic. J. Am. Coll. Cardiol..

[2] Chugh S.S., Reinier K., Teodorescu C., Evanado A., Kehr E., Al Samara M., Mariani R., Gunson K., Jui J. (2008). Epidemiology of Sudden Cardiac Death: Clinical and Research Implications. Prog. Cardiovasc. Dis..

[3] Berdowski J., Berg R.A., Tijssen J.G.P., Koster R.W. (2010). Global incidences of out-of-hospital cardiac arrest and survival rates: Systematic review of 67 prospective studies. Resuscitation.

[4] Myerburg R.J., Junttila M.J. (2012). Sudden Cardiac Death Caused by Coronary Heart Disease. Circulation.

[5] Kaikkonen K.S., Kortelainen M.L., Linna E., Huikuri H.V. (2006). Family History and the Risk of Sudden Cardiac Death as a Manifestation of an Acute Coronary Event. Circulation.

[6] Stecker E.C., Reinier K., Marijon E., Narayanan K., Teodorescu C., Uy-Evanado A., Gunson K., Jui J., Chugh S.S. (2014). Public Health Burden of Sudden Cardiac Death in the United States. Circ. Arrhythmia Electrophysiol..

[7] Burke A.P., Farb A., Malcom G.T., Liang Y.H., Smialek J., Virmani R. (1997). Coronary Risk Factors and Plaque Morphology in Men with Coronary Disease Who Died Suddenly. N. Engl. J. Med..

[8] Myerburg R.J., Goldberger J.J. (2017). Sudden Cardiac Arrest Risk Assessment: Population Science and the Individual Risk Mandate. JAMA Cardiol..

[9] Winkel B.G., Holst A.G., Theilade J., Kristensen I.B., Thomsen J.L., Ottesen G.L., Bundgaard H., Svendsen J.H., Haunsø S., Tfelt-Hansen J. (2011). Nationwide study of sudden cardiac death in persons aged 1–35 years. Eur. Heart J..

[10] Waldmann V., Karam N., Bougouin W., Sharifzadehgan A., Dumas F., Narayanan K., Spaulding C., Cariou A., Jouven X., Marijon E. (2019). Burden of Coronary Artery Disease as a Cause of Sudden Cardiac Arrest in the Young. J. Am. Coll. Cardiol..

[11] Oving I., De Graaf C., Karlsson L., Jonsson M., Kramer-Johansen J., Berglund E., Hulleman M., Beesems S.G., Koster R.W., Olasveengen T.M. (2020). Occurrence of shockable rhythm in out-of-hospital cardiac arrest over time: A report from the COSTA group. Resuscitation.

[12] Sasson C., Rogers M.A.M., Dahl J., Kellermann A.L. (2010). Predictors of Survival From Out-of-Hospital Cardiac Arrest: A Systematic Review and Meta-Analysis. Circ. Cardiovasc. Qual. Outcomes.

[13] Weisfeldt M.L., Becker L.B. (2002). Resuscitation After Cardiac Arrest: A 3-Phase Time-Sensitive Model. JAMA.

[14] Link M.S., Berkow L.C., Kudenchuk P.J., Halperin H.R., Hess E.P., Moitra V.K., Neumar R.W., O’Neil B.J., Paxton J.H., Silvers S.M. (2015). Part 7: Adult Advanced Cardiovascular Life Support: 2015 American Heart Association Guidelines Update for Cardiopulmonary Resuscitation and Emergency Cardiovascular Care. Circulation.

[15] Kudenchuk P.J., Brown S.P., Daya M., Rea T., Nichol G., Morrison L.J., Morrison L.J., Leroux B., Vaillancourt C., Wittwer L. (2016). Amiodarone, Lidocaine, or Placebo in Out-of-Hospital Cardiac Arrest. N. Engl. J. Med..

[16] Soar J., Böttiger B.W., Carli P., Couper K., Deakin C.D., Djärv T., Lott C., Olasveengen T., Paal P., Pellis T. (2021). European Resuscitation Council Guidelines 2021: Adult advanced life support. Resuscitation.

[17] Wang C., Gao Y., Liu Y., Yao Y., Li C., Li Q., Chai Y. (2022). Analysis of factors influencing cardiopulmonary resuscitation and survival outcome in adults after in-hospital cardiac arrest: A retrospective observational study. Chin. Med. J..

[18] Saghafinia M., Motamedi M.H., Piryaie M., Rafati H., Saghafi A., Jalali A., Madani S.J., Kolahdehi R.B. (2010). after in-hospital cardiopulmonary resuscitation in a major referral center. Saudi. J. Anaesth..

[19] Sans Roselló J., Vidal-Burdeus M., Loma-Osorio P., Pons Riverola A., Bonet Pineda G., El Ouaddi N., Aboal J., Solé A.A., Scardino C., García-García C. (2022). Impact of age on management and prognosis of resuscitated sudden cardiac death patients. IJC Heart Vasc..

[20] Zanders R., Druwé P., Van Den Noortgate N., Piers R. (2021). The outcome of in- and out-hospital cardiopulmonary arrest in the older population: A scoping review. Eur. Geriatr. Med..

[21] Connolly M.S., Goldstein Pcp J.P., Currie M., Carter A.J.E., Doucette S.P., Giddens K., Allan K.S., Travers A.H., Ahrens B., Rainham D. (2022). Urban-Rural Differences in Cardiac Arrest Outcomes: A Retrospective Population-Based Cohort Study. CJC Open.

[22] Ro Y.S., Shin S.D., Song K.J., Lee E.J., Kim J.Y., Ahn K.O., Chung S.P., Kim Y.T., Hong S.O., Choi J.-A. (2013). A trend in epidemiology and outcomes of out-of-hospital cardiac arrest by urbanization level: A nationwide observational study from 2006 to 2010 in South Korea. Resuscitation.

[23] Andersen L.W., Grossestreuer A.V., Donnino M.W. (2018). “Resuscitation time bias”—A unique challenge for observational cardiac arrest research. Resuscitation.

[24] Reynolds J.C., Grunau B.E., Rittenberger J.C., Sawyer K.N., Kurz M.C., Callaway C.W. (2016). Association Between Duration of Resuscitation and Favorable Outcome After Out-of-Hospital Cardiac Arrest: Implications for Prolonging or Terminating Resuscitation. Circulation.

[25] Wongtanasarasin W., Srisurapanont K., Nishijima D.K. (2023). How Epinephrine Administration Interval Impacts the Outcomes of Resuscitation during Adult Cardiac Arrest: A Systematic Review and Meta-Analysis. JCM.

[26] Park S., Lee S.W., Han K.S., Lee E.J., Jang D.H., Lee S.J., Kim S.J., Korean Cardiac Arrest Research Consortium (KoCARC) Investigators (2022). Optimal cardiopulmonary resuscitation duration for favorable neurological outcomes after out-of-hospital cardiac arrest. Scand. J. Trauma. Resusc. Emerg. Med..

[27] Bai Z., Wang L., Yu B., Xing D., Su J., Qin H. (2023). The success rate of cardiopulmonary resuscitation and its correlated factors in patients with emergency prehospital cardiac arrest. Biotechnol. Genet. Eng. Rev..

[28] Sigal A.P., Sandel K.M., Buckler D.G., Wasser T., Abella B.S. (2019). Impact of adrenaline dose and timing on out-of-hospital cardiac arrest survival and neurological outcomes. Resuscitation.

[29] Rea T., Kudenchuk P.J. (2021). Death by COVID-19: An Open Investigation. JAHA.

[30] Bharmal M., DiGrande K., Patel A., Shavelle D.M., Bosson N. (2023). Impact of Coronavirus Disease 2019 Pandemic on Cardiac Arrest and Emergency Care. Heart Fail. Clinics..

[31] Ball J., Nehme Z., Bernard S., Stub D., Stephenson M., Smith K. (2020). Collateral damage: Hidden impact of the COVID-19 pandemic on the out-of-hospital cardiac arrest system-of-care. Resuscitation.

[32] Stankovic N., Høybye M., Holmberg M.J., Lauridsen K.G., Andersen L.W., Granfeldt A. (2021). Factors associated with shockable versus non-shockable rhythms in patients with in-hospital cardiac arrest. Resuscitation.

[33] Bielski K., Makowska K., Makowski A., Kopiec T., Gasecka A., Malecka M., Pruc M., Rafique Z., Peacock F.W., Denegri A. (2021). Impact of COVID-19 on in-hospital cardiac arrest outcomes: An updated meta-analysis. Cardiol J..

[34] Mir T., Sattar Y., Ahmad J., Ullah W., Shanah L., Alraies M.C., Qureshi W.T. (2022). Outcomes of in-hospital cardiac arrest in COVID-19 patients: A proportional prevalence meta-analysis. Cathet Cardio Interv..

[35] Yuksen C., Phattharapornjaroen P., Kreethep W., Suwanmano C., Jenpanitpong C., Nonnongku R., Sittichanbuncha Y., Sawanyawisuth K. (2020). Adrenaline use as a poor predictor for the return of spontaneous circulation among victims of out-of-hospital cardiac arrest according to a national emergency medical services database. Turk. J. Emerg. Med..

[36] Li Z., Xing J. (2023). A model for predicting return of spontaneous circulation and neurological outcomes in adults after in-hospital cardiac arrest: Development and evaluation. Front. Neurol..

[37] Shinada K., Matsuoka A., Miike T., Koami H., Sakamoto Y. (2024). Effects of physician-present prehospital care in patients with out-of-hospital cardiac arrest on return of spontaneous circulation: A retrospective, observational study in Saga, Japan. Health Sci. Rep..

[38] Karakasis P., Fragakis N., Patoulias D., Theofilis P., Kassimis G., Karamitsos T., MRizzo M. (2024). Effects of Glucagon-Like Peptide 1 Receptor Agonists on Atrial Fibrillation Recurrence After Catheter Ablation: A Systematic Review and Meta-analysis. Adv Ther..

